# Characterization of thrombin/factor Xa inhibitors in Rhizoma Chuanxiong through UPLC-MS-based multivariate statistical analysis

**DOI:** 10.1186/s13020-020-00376-0

**Published:** 2020-08-31

**Authors:** Yi-Yao Yang, Zhao-Yu Wu, Fang-Bo Xia, Hao Zhang, Xu Wang, Jian-Li Gao, Feng-Qing Yang, Jian-Bo Wan

**Affiliations:** 1 School of Chemistry and Chemical Engineering, Chongqing University, Chongqing, 401331 People’s Republic of China; 2 State Key Laboratory of Quality Research in Chinese Medicine, Institute of Chinese Medical Sciences, University of Macau, Taipa, Macao SAR People’s Republic of China; 3 Academy of Chinese Medical Sciences, Zhejiang Chinese Medical University, Hangzhou, 310053 Zhejiang People’s Republic of China; 4 College of Pharmacy, Chengdu University of Traditional Chinese Medicine, Chengdu, Sichuan China

**Keywords:** Chuanxiong, Thrombin, Factor Xa, Enzyme inhibitor, Multivariate statistical analysis, Molecular docking

## Abstract

**Background:**

The dry root and rhizome of *Ligusticum chuanxiong* Hort., or Chuanxiong, has been used as a blood-activating and stasis-removing traditional Chinese medicine for 1000 years. Our previous studies have shown the inhibitory activity on platelet and thrombin (THR) of Chuanxiong. THR and factor Xa (FXa) play significant roles in the coagulation cascade and their inhibitors are of valuable in the treatment of thromboembolic diseases. The aim of the present study is to screen THR and FXa inhibitors from Chuanxiong.

**Methods:**

Four extracts [ethyl acetate (EA), butanol (BA) and remained extract (RE) from 75% ethanol extract, and water extract (WE)] of Chuanxiong were prepared, and their THR/FXa inhibitory activities were assessed in vitro. Following silica-gel column chromatography (SC), the active EA extract and BA extract was further partitioned, respectively. Their active fractions (EA-SC1 to EA-SC5; BA-SC1 to BA-SC5) were obtained and analyzed by LC–MS. After modeling by the principal component analysis (PCA) and orthogonal partial least squares discriminate analysis (OPLS-DA), the specific marker compounds were predicted and identified. Their enzyme inhibitory was assessed in vitro and interactions with THR/FXa were investigated by molecular docking analysis.

**Results:**

Chuanxiong EA extract showed strong activity against THR and BA extract was more effective in inhibiting FXa activity, and their fractions exhibited obvious difference in enzyme inhibitory activity. Furthermore, marker compounds **a**–**h** were predicted by PCA and OPLS-DA, and their chemical structures were identified. Among them, senkyunolide A, *Z*-ligustilide, ferulic acid and senkyunolide I (IC_50_ was determined as 0.77 mM) with potential THR inhibitory activity, as well as isochlorogenic acid A with FXa inhibitory activity were screened out. It was found that the four components could interact with the active site of THR, and the binding energy was lower than − 5 kcal/mol. Isochlorogenic acid A were bound to the active site of FXa, and the binding energy was − 9.39 kcal/mol. The IC_50_ was determined as 0.56 mM.

**Conclusions:**

THR/FXa inhibitory components in different extracts of Chuanxiong were successfully characterized by the method of enzyme inhibition activity assays with ultra performance liquid chromatography-quadrupole time of flight mass spectrometry-based multivariate statistical analysis.

## Background

Thrombin (THR) and factor Xa (FXa) are the members of the serine protease family. As the pivotal enzyme in the blood coagulation processes, FXa act as a catalyst in the THR production by activating prothrombin without existing THR affected [1, 2], and THR catalyze the conversion of fibrinogen into insoluble strands of fibrin, as well as stimulate and recruit platelets to the injured site [3]. Due to the importance during blood coagulation cascade, FXa and THR potentially have emerged as attractive targets for new anticoagulants to treat thrombotic diseases. On the other hand, there have been several literature focusing on the study of THR/FXa inhibitory activity of natural products, which include polypeptides [4–7], polyphenols [8, 9], saponins [10] and other compounds [11–13]. Rhizoma Chuanxiong, the dried root of *Ligusticum chuanxiong* Hort. (Umbelliferae), namely Chuanxiong in Chinese, is a famous blood circulation promoting medicine and is one of the clinically used traditional Chinese medicine (TCM) in protecting cardiovascular system. As genuine medicinal material, Chuanxiong is mainly distributed in Sichuan province [14]. Its major chemical components include phthalide lactones, alkaloids, phenolic acids and other constituents [15]. A large body of studies has shown that Chuanxiong possesses multifarious pharmacological effects, including protective effects on neuron [16], heart [17], liver [18] and kidney [19], as well as antioxidation [20, 21], anti-inflammation [22–24], etc. In our previous studies, Chuanxiong extracts had inhibitory effects on platelet aggregation [25] and THR activity [12], while there are few studies on its effects on THR and FXa so far. It is of reasonable to screen THR/FXa inhibitors from Chuanxiong.

Natural products, especially TCMs, are valuable sources of active components for the discovery of novel clinical drug candidates [26–28]. Various techniques to characterize bioactive components from natural products had been reported. The multivariate statistical analysis method can analyze huge amount data generated from liquid chromatography paired with mass spectrometry (LC-MS), and rapidly distinguish the chemical difference among different sample groups [29]. This method had been adopted several times in natural product research for screening of bioactive components [30] or quality control markers [31], studying mechanisms of TCM processing [32] and compatibility [33]. The pharmacological ingredients from the natural products can be efficiently determined by multivariate statistical analysis when combined with bioactivity analysis [34]. Recently, this method has been proved practical and effective in identifying antiplatelet components of edible *Citrus limon* [35], for the analysis of antidiabetic compounds from TCM formula Ge-Gen-Qin-Lian decoction [36] and for the screening of potential THR/FXa inhibitors from Danshen [37].

In this study, ultra performance liquid chromatography-quadrupole time of flight mass spectrometry (UPLC-QTOF-MS) combined with enzyme inhibition activity assays were carried out for analyzing different Chuanxiong fractions. Principal component analysis (PCA) was used to reduce the dimensionality of MS data. Then, orthogonal partial least squares discriminant analysis (OPLS-DA) models were fitted to find out the differential marker compounds based on activity assay results, and their structures were identified. Furthermore, the interaction behaviors between the selected compounds and the enzyme were elucidated by molecular docking analysis. Finally, the enzyme inhibition activities of the marker compounds were evaluated.

## Materials and methods

### Plant material

Crude drug of Rhizoma Chuanxiong was purchased from Chongqing Xinhu Pharmacy Co., Ltd. (Chongqing, China), and was authenticated by Professor Feng-Qing Yang (School of Chemistry and Chemical Engineering, Chongqing University). The voucher specimen (No. CX2019033001) was deposited at the Pharmaceutical Engineering Laboratory in the School of Chemistry and Chemical Engineering, Chongqing University, Chongqing, China.

### Chemicals and reagents

*Z*-ligustilide, senkyunolide A and isochlorogenic acid A were purchased from Purechem-standard Co., Ltd. (Chengdu, China). Senkyunolide I, isochlorogenic acid B and isochlorogenic acid C were purchased from PUSH Bio-technology Co., Ltd. (Chengdu, China). Ferulic acid, dopamine hydrochloride and rivaroxaban were obtained from Aladdin Chemistry Co., Ltd. (Shanghai, China). Dimethyl sulphoxide (DMSO) and Tris (hydroxymethyl) aminomethane (Tris) were obtained from Sangon Biotech Co., Ltd. (Shanghai, China). Argatroban was purchased from Harvey-bio Co., Ltd. (Beijing, China). FXa and two chromogenic substrates S-2238 and S-2765 were products of Adhoc International Technologies Co., Ltd. (Beijing, China), and THR was bought from Sigma-Aldrich (St Louis, USA). HPLC-grade acetonitrile and formic acid were obtained from Thermo Fisher Scientific Co., Ltd. (China). HPLC-grade methanol were obtained from Shanghai Tedia Scientific Co., Ltd. (Shanghai, China). All of the experimental water was purified by a water-purification system (ATSelem 1820A, Antesheng Environmental Protection Equipment Co., Ltd., Chongqing, China). Ethanol, butanol (BA), ethyl acetate (EA), petroleum ether (PE), sodium hydroxide (NaOH) and hydrochloric acid (HCl) were of analytical grade and purchased from Chron Chemicals Co., Ltd. (Chengdu, China).

The Tris-HCl buffer was prepared by adding 1 M HCl to 10 mM Tris solution (pH 8.0). All samples were prepared in Tris-HCl buffer (10 mM, pH 8.0) with cosolvent DMSO, and diluted to the required concentrations for THR/FXa inhibitory assay, which were stored at 4 °C and shielded from light before use. The stock solution of THR was prepared in Tris-HCl buffer (10 mM, pH 8.0) with the enzyme activity of 500 U/mL, and stored at − 20 °C. FXa was also dispensed in Tris-HCl buffer (10 mM, pH 8.0) with the enzyme activity of 0.5 IU/mL and stored at 4 °C.

### Preparation of sample extracts

After comminution, 100 g of Chuanxiong powder was extracted with 800 mL 75% ethanol (1:8, w/v) in a 2 L glass-stoppered conical flask on water bath at 80 °C for 1 h; then the extract was filtered, and the residue was collected and extracted with the above process for twice. The three filtrates were combined and concentrated in a rotary evaporator (ZFQ 85 A, Shanghai Medical Instrument Special Factory, Shanghai, China) at 45 °C. After removing ethanol completely, the concentrate were degreased with petroleum ether (2:1, v/v), and further subjected to liquid–liquid partitioning to afford EA (2:1, v/v), BA (1:1, v/v) soluble extracts as well as the remained extract (RE). After removing the solvent from each solution, the extracts were obtained by reduced pressure distillation and vacuum dry method (DZF-6050, Shanghai Jing Hong Laboratory Instrument Co., Ltd., Shanghai, China). In addition, the residues was extracted twice with 600 mL water (1:6, w/v) on water bath at 80 °C. The two filtrates were combined and evaporated, and further vacuum-dried to obtain the water extract (WE). Total 200 g Chuanxiong powder was used for extraction.

### In vitro THR/FXa inhibitory activity assays

THR/FXa inhibitory activity assays were performed on an Agilent 7100 3^D^ capillary electrophoresis (CE) system (Agilent Technologies, Palo Alto, CA, USA), which was equipped with a diode array detector (DAD) and Agilent ChemStation software. All of the experimental procedures were implemented according to literature with minor modifications [38]. The preparation process of immobilized enzyme microreactor (IMER) is described as follows. A new bare fused-silica capillary (i.d. 75 μm, purchased from Yongnian Ruifeng Chromatographic Device Co., Ltd., Hebei, China) were pretreated with NaOH (1 M) and deionized water for 15 min and 10 min, respectively. An automated program was set to prepare the IMER: The dopamine solution (2 mg/mL) was injected into the capillary with a voltage of + 10 kV for 10 s, stayed for 30 min, and then washed out the free dopamine using running buffer (10 mM Tris–HCl buffer solution, pH 8.0) with a pressure of − 100 mbar for 90 s. Then, the 125 U/mL THR solution (or 0.5 IU/mL FXa solution) was introduced into the capillary with a voltage of + 10 kV for 10 s, kept for 30 min; and then flushed by running buffer with a pressure of − 100 mbar for 90 s to wash out free enzyme. The prepared IMER could be immediately used for enzyme inhibitory activity assay. The temperature of the capillary cartridge was maintained at 25 °C during the CE analysis. The enzyme inhibitory activity assays were carried out by a reaction that the 2.5 mg/mL substrate solution (S-2238 for THR assays, S-2765 for FXa assays) with/without inhibitors were injected into the IMER inlet by applying a voltage of + 10 kV for 10 s and incubated for 60 s to trigger amidolytic reaction. In order to detect the product p-nitroaniline, the voltage of + 25 kV was applied to separate the reaction mixtures and the detection wavelength was set at 405 nm. The inhibition percentage was calculated by the formula:

1 $${\text{Inhibition percentage}}\left( {\text{\% }} \right) = \left( {1 - \frac{{A_{sample} }}{{A_{blank} }}} \right) \times 100$$

where $A_{blank}$ and $A_{sample}$ represent the peak area of product yielded by enzymatic reaction of the blank and sample group, respectively. All assays were performed in triplicate and the % of inhibition was the mean of three observations.

### HPLC-DAD analysis

HPLC analysis was performed on an Agilent 1260 Series liquid chromatography system (Agilent Technologies, Palo Alto, California, USA) which was equipped with a vacuum degasser, a binary pump, an autosampler, and a diode array detector (DAD), controlled by an Agilent ChemStation software. An Agilent ZORBAX SB-C18 column (250 × 4.6 mm i.d., 5 µm) preceded by ZORBAX SB-C18 guard column (12.5 × 4.6 mm i.d., 5 μm) was used for the separation. The mobile phase consisted of solvent A (0.1% formic acid in water) and solvent B (methanol) with a gradient elution program, which was programmed as follows: 0–20 min, 20–35% B; 20–32 min, 35–43% B; 32–42 min, 43–60% B; 42–47 min, 60–80% B; 47–52 min, 80% B; 52–57 min, 80–20% B; 57–62 min, 20% B. The solvent flow rate was set at 0.6 mL/min. The detection wavelength was set at 273 nm, and the column temperature was maintained at 35 °C and the injection volume was 10 µL.

### UPLC-QTOF-MS analysis

A Waters ACQUITY™ UPLC system coupled with a QTOF SYNAPT G2-Si high-definition mass spectrometer (Waters, Manchester, UK) equipped with an electrospray ionization (ESI) source, was used for the LC–MS analysis and identification. The LC conditions were implemented according to literatures with minor modifications [39]. Chromatographic separations were achieved on an ACQUITY™ BEH C18 column (100 mm × 2.1 mm i.d., 1.7 μm) at 45 °C. The mobile phase was composed of 0.1% aqueous formic acid solution (A) and acetonitrile (B) at the flow rate of 0.4 mL/min. The gradient program were employed as fellows: 0–6 min, 5–50% B; 6–12 min, 50–60% B; 12–16 min, 60–85% B; 16–17 min, 85–100% B; 17–20 min, 100% B; 20–20.5 min, 100–5% B; 20.5–23 min, isocratic 5% B. The injection volume was 3 μL. The LC eluent was introduced to a QTOF MS was operated in positive ion mode. The MS parameters were set as follows: capillary voltage, 3.0 kV; sample cone voltage, 40 V; source temperature, 150 °C; desolvation temperature, 500 °C; collision energy ramp, 20–40 V. The MS data were acquired by MS^E^ scan mode with a mass range from *m*/*z* 100 to *m*/*z* 1500.

### Data processing and multivariate analysis

The raw LC–MS data of Chuanxiong samples were extracted and processed by Progenesis QI software (Waters Corporation, Milford, MA, USA). After peak recognition, alignment and integration, the intensity of each ion was normalized across samples according to total intensity of each chromatogram. A resultant three-dimensional dataset composing of the sample code, peak name (*t*_R_-*m*/*z* pair) and ion intensity, was generated. After data pre-treatment by mean-centered and *pareto*-scale methods, multivariate statistical analysis, including PCA and OPLS-DA, were conducted by SIMCA-P^+^ 13.0 Software (Umetrics, Umeå, Sweden).

### In silico molecular docking of THR/FXa and identified potential active compounds

The purpose of in silico molecular docking study is to validate the binding energy between enzymes and small molecular compounds, which were carried out by Auto Dock 4.2 program (The Scripps Research Institute, La Jolla, CA, USA). The docking operation was performed according to the following steps. Firstly, prepare the file of receptor protein. The X-ray co-crystal structure file of THR-argatroban complex (PDB code = 1DWC [40]) and FXa-rivaroxaban complex (PDB code = 2W26 [41]) were acquired from Protein Data Bank database. Next, the co-crystallized ligand, water were removed, and polar hydrogen atoms were added. Then, the 3D chemical structures of small molecular compounds were drawn by Chem Office and minimized energy with outputting in PDB format. Finally, Lamarckian genetic algorithm (LGA) was employed and the number of GA runs was equal to 50 for finding the most favorable ligand binding orientations. The 2D interaction diagrams of optimum conformation after docking was generated by Discovery Studio 4.5 (Dassault Systèmes BIOVIA, San Diego, CA, USA) to observe the interaction between molecular compounds and the residues of enzyme.

## Results

### Bioactivity-guided fractionation

**Fig. 1:**
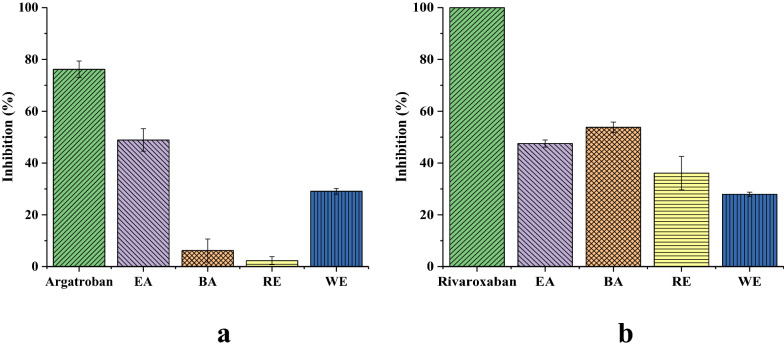
Inhibition percentage (%) of different fractions of Chuanxiong crude extract on the THR (**a**) and FXa (**b**). Data are shown as mean ± SD from three independent experiments (n = 3)

The activity evaluation tests against THR/FXa of Chuanxiong different polar extracts (EA, BA, RE and WE extracts, 1.5 mg/mL) and each positive control, argatroban and rivaroxaban (0.5 mg/mL) were employed, and the results were expressed in Fig. 1. The EA extract was more effective in inhibiting THR activity, and the BA extract showed the strongest inhibitory activity toward FXa, which were prioritized for further fractionation.

**Fig. 2:**
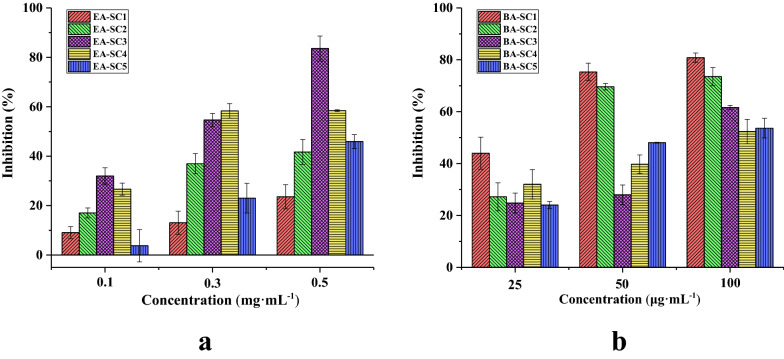
Inhibition percentage (%) of silica gel fractions of Chuanxiong EA extract on THR (**a**), and BA extract on FXa (**b**). Data are shown as mean ± SD from three independent experiments (n = 3)

The EA extract (4.16 g) was applied to normal silica gel column chromatography (SC), and eluted with PE-EA (40:1 to 1:20, v/v) followed by 100% EA. HPLC analysis was applied to recombine the obtained fractions to give five fractions (EA-SC1 to EA-SC5). The BA extract (4.35 g) was eluted with 100% EA, followed by EA-ME (50:1 to 1:3, v/v) and 100% ME in sequence. HPLC analysis was also applied and five fractions were yielded (BA-SC1 to BA-SC5). THR inhibitory activity of the five EA fractions was measured (Fig. 2a). Fraction EA-SC3 exhibited the strongest inhibitory effect, and fractions EA-SC2, EA-SC4 and EA-SC5 were shown moderate activity, while the inhibition percentage (%) of THR by EA-SC1 was consistently low compared to the other fractions. As shown in Fig. 2b, the results of FXa inhibitory activity assays among BA fractions indicated that these five fractions could be grouped into two classifications: the most active (BA-SC1 and BA-SC2) and moderate active (BA-SC3 to BA-SC5) groups.

### Multivariate statistical analysis of active compounds from different fractions

**Fig. 3:**
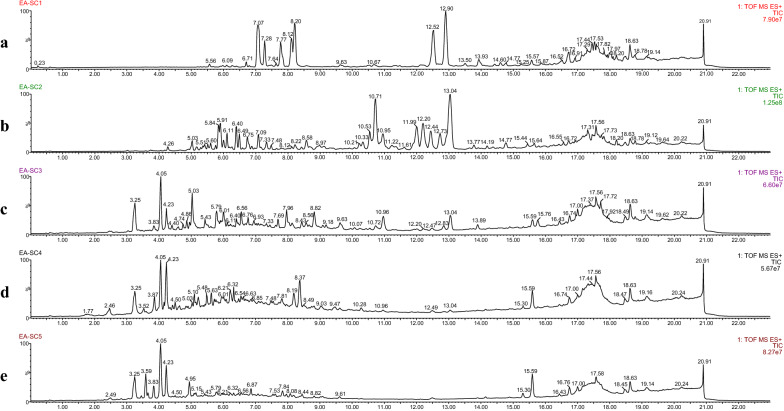
Total ion chromatograms of five Chuanxiong fractions from EA extract in positive mode. **a** EA-SC1, **b** EA-SC2, **c** EA-SC3, **d** EA-SC4 and **e** EA-SC5

**Fig. 4:**
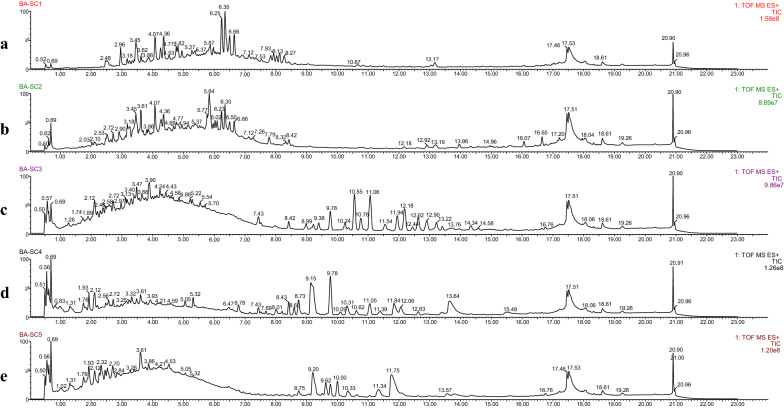
Total ion chromatograms of five Chuanxiong fractions from BA extract in positive mode. **a** BA-SC1, **b** BA-SC2, **c** BA-SC3, **d** BA-SC4 and **e** BA-SC5

The Chuanxiong fractions, separated by silica column, was subjected to LC–MS analysis in order to conduct an investigation of the differences in their chemical profiles and screening of active marker compounds. The representative total ion chromatographs of five EA fractions (EA-SC1 to EA-SC5) were illustrated in Fig. 3, and that of five BA fractions (BA-SC1 to BA-SC5) were shown in Fig. 4. This two-processed LC–MS data matrix was generated by Progenesis QI and then was separately imported into SIMCA for unsupervised PCA analysis and supervised OPLS-DA analysis.

**Fig. 5:**
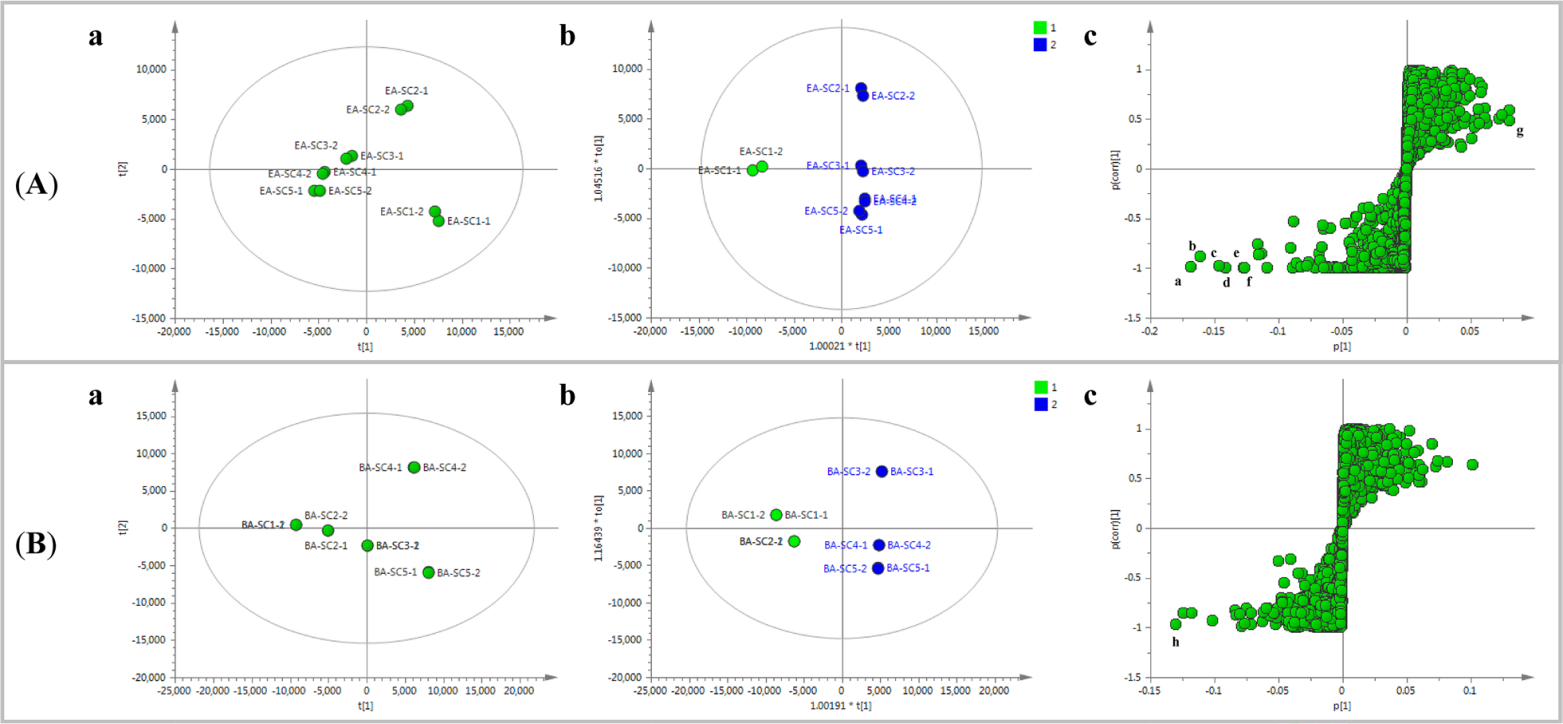
Chemometric analysis of Chuanxiong EA fractions (**A**) and BA fractions (**B**). Score plot from PCA (**a**), Score plot (**b**) and S-plot (**c**) from OPLS-DA, Ellipse: Hotelling’s 95% confidence

To provide visualization of the differences among the Chuanxiong EA fractions, unsupervised PCA analysis was conducted. The score plot (Fig. 5Aa) showed preferably discriminative distribution, and the PCA map could be mainly divided into two clusters: EA-SC1, EA-SC2 to EA-SC5. A correlation was found that this cluster was similar to total ion chromatography of each EA fraction (Fig. 3), which is also consistent with the THR inhibitor activity (Fig. 2). The values of the PCA model fit parameters were 0.782 of *R*^2^*X* (cum) and 0.622 of *Q*^2^ (cum) and all the samples fell well inside the 95% confidence interval, indicating a good PCA model. Subsequently, to explore the potential active marker compounds, supervised OPLS-DA analysis was employed to group the Chuanxiong EA fractions in a binary classification as the active and less active groups. The OPLS-DA score plot was presented in Fig. 5Ab, and the five fractions are clearly distinguished and could be classified as active (EA-SC2 to EA-SC5) and less active (EA-SC1). The model fit parameters *R*^2^*Y* (cum) and *Q*^2^ (cum) were 0.996 and 0.984, respectively, and all the observations fell within the Hotelling T2 (0.95) ellipse, which suggested that the OPLS-DA model (M_THR_) exhibited good fitting and predictability [42]. In the S-plot (Fig. 5Ac), the ion points far away from the centre (the corner of “S”-shaped curve) indicated a larger contribution to the classification of the samples. Marker ions **a**–**g** were selected with high variable importance in the projection (VIP) scores (VIP > 1). The detailed information was listed in Table 1.

**Table 1:** Detailed information of eight marker compounds obtained from OPLS-DA S-plot in Chuanxiong fractions

Marker compounds	t_R_ (min)–*m*/*z*	Ions	VIP	Formula (neutral form)	Identification
a*	7.07–193	[M+H]^+^	16.09	C_12_H_16_O_2_	Senkyunolide A
b*	12.90–783	[2M+Na]^+^	15.41	C_24_H_28_O_4_	Ligustilide dimer
c*	12.52–191	[M+H-ligustilide]^+^	13.97	C_24_H_28_O_4_	Ligustilide dimer
d*	8.22–191	[M+H]^+^	13.52	C_12_H_14_O_2_	*Z*-Ligustilide
e*	7.77–191	[M+H]^+^	12.19	C_12_H_14_O_2_	Ligustilide isomer
f*	8.12–177	[M+H-H_2_O]^+^	12.10	C_10_H_10_O_4_	Ferulic acid
g*	4.05–207	[M+H-H_2_O]^+^	8.00	C_12_H_16_O_4_	Senkyunolide I
h^#^	3.45–499	[M+H-H_2_O]^+^	14.17	C_25_H_24_O_12_	Isochlorogenic acid A

* Represents the marker screened from M_THR_

^#^Represents the marker screened from M_FXa_

The same procedure was employed to discover potential marker compounds from Chuanxiong BA fractions. PCA analysis were employed for investigating the similarity of the constituent profiles of Chuanxiong BA fractions. The values of the PCA model fit parameters were 0.995 for *R*^2^*X* (cum) and 0.985 for *Q*^2^ (cum) and all the samples fell well inside the 95% confidence interval. As shown in Fig. 5Ba, in the PCA scores plot of Chuanxiong BA fractions, BA-SC1 and BA-SC2 were separated into a cluster distinct from other fractions, which exhibited a trend similar to total ion chromatography of each BA fraction and were observed corresponding with the results of FXa inhibitory activity assays. Then, the OPLS-DA model (M_FXa_) was fitted and showed good fitness and predictability with *Q*^2^ (cum) = 0.963, *R*^2^*Y* (cum) = 0.985. All the observations fell within the Hotelling T2 (0.95) ellipse. As presented in Fig. 5Bb, the OPLS-DA score plot illustrated that the five fractions could be clearly distinguished and classified as active (BA-SC1, BA-SC2) and less active (BA-SC3 to BA-SC5). In the S-plot (Fig. 5Bc), marker ion **h** in the extreme lower left quadrant was selected.

### Mass fragmentation analysis of marker compounds

**Fig. 6:**
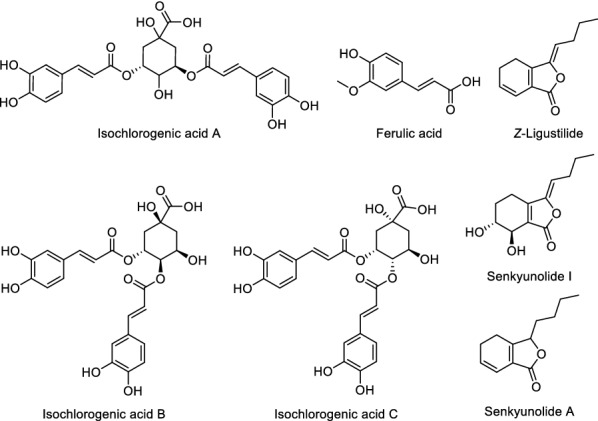
Chemical structures of the marker compounds prioritized from Chuanxiong extract and the compounds with similar structure

The identification of chemical markers **a**–**h** was further investigated, MS^2^ data verification was conducted with the assist of reported literature [12, 39, 43–46]. Five compounds, including senkyunolide A (**a**), *Z*-ligustilide (**d**), ferulic acid (**f**), senkyunolide I (**g**) and isochlorogenic acid A (**h**), were tentatively identified, and their chemical structures were shown in Fig. 6.

Marker **a** displayed [M+H]^+^ ion at *m*/*z* 193 and [M+Na]^+^ ion at *m*/*z* 215. The product ions were obtained at *m*/*z* 175 for [M+H-H_2_O]^+^, *m*/*z* 147 for [M+H-H_2_O-CO]^+^, *m*/*z* 137 for [M+H-C_4_H_8_]^+^, *m*/*z* 119 for [M+H-H_2_O-CO-C_2_H_4_]^+^ and *m*/*z* 105 for [M+H-H_2_O-CO-C_3_H_6_]^+^. Therefore, marker **a** was identified as senkyunolide A [39, 43]. Marker **d** yielded [M+H]^+^ ion at *m*/*z* 191, which produced fragment ions [M+H-H_2_O]^+^, [M+H-H_2_O-CO]^+^, [M+H-H_2_O-CO-C_2_H_4_]^+^, [M+H-H_2_O-CO-C_2_H_6_]^+^ and [M+H-H_2_O-CO-C_3_H_4_]^+^ at *m*/*z* 173, 145, 117, 115, 105. Marker **e** and peaks in 7.28 min and 7.84 min showed same ion fragments as marker **d**, which were isomers of butylphthalide/*E*-ligustilide/*Z*-ligustilide. According to the peak occurrence sequence and literature [39], marker **d** was identified as *Z*-ligustilide. Marker **f** showed peak of [M+H]^+^ ion at *m*/*z* 195 and [M+Na]^+^ ion at *m*/*z* 217. The product ions were obtained at *m*/*z* 177 for [M + H-H_2_O]^+^ and *m*/*z* 149 for [M + H-HCOOH]^+^. Therefore, marker **f** was identified as ferulic acid [43–45]. Marker **g** gave [M+Na]^+^ ion at *m*/*z* 247. Ions at *m*/*z* 207, *m*/*z* 189 and *m*/*z* 161 were also detected, which were formed by sequential losses of H_2_O, H_2_O and CO from [M+H]^+^ ion. Therefore, marker **g** was identified as senkyunolide I [39, 44].

Marker **h** exhibited typical fragment ion at *m*/*z* 499 by the loss of the group of H_2_O from [M+H]^+^ ion at *m*/*z* 517. The [caffeoyl + H]^+^ ion at *m*/*z* 163 were also found, which were deduced to be formed by the loss of caffeoyl and quinic acid from [M+H]^+^ ion. In order to further confirming its structure, the MS data in the negative ion mode was recorded. The typical and major fragment ions were generated by the loss of caffeoyl and H_2_O from [M-H]^−^ ion (*m*/*z* 515), such as [M-H-caffeoyl]^−^ *m*/*z* 353, [M-H-caffeoyl-H_2_O]^−^ *m*/*z* 335 and [M-H-2caffeoyl-H_2_O]^−^ *m*/*z* 191. With the reference compounds and literature [12, 46], marker **h** was identified as isochlorogenic acid A.

### Molecular docking analysis of THR/FXa and identified potential active compounds

**Fig. 7:**
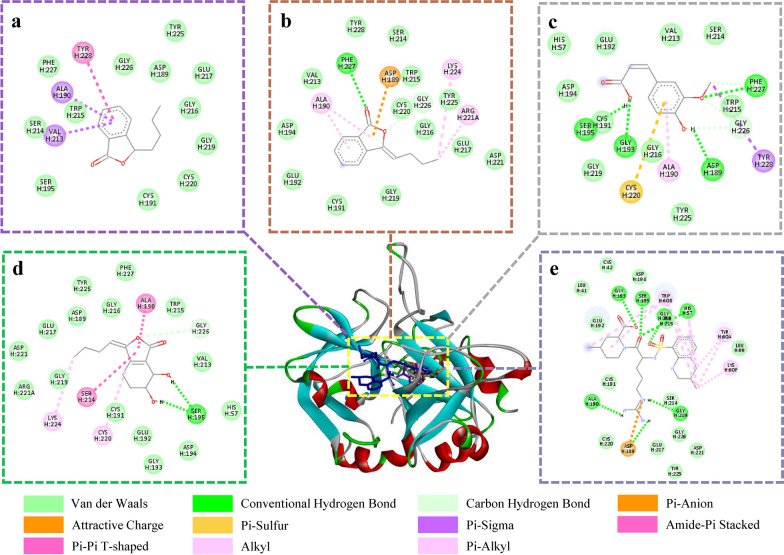
Binding mode and interaction between four screened inhibitors and the THR catalytic site. **a** Senkyunolide A, **b** *Z*-Ligustilide, **c** Ferulic acid, **d** Senkyunolide I, **e** Argatroban

Molecular docking can be used to study the binding mechanism of compounds interacting with the active site of proteins. The docking energy and binding residues of four markers (from Chuanxiong EA fractions) with THR active site were gathered in Table 2. The active sites of THR have four binding pockets [47]: S1 pocket (specificity pocket), S2 pocket (proximal pocket), S3 pocket, and S4 pocket (aryl binding pocket). For the docking with THR (Fig. 7), it was observed that four marker compounds could insert into the catalytic active pocket of THR like original ligand argatroban via multifarious interactions such as hydrogen bond and van der Waals, etc. The main part of argatroban interacted with S2 pocket, and its guanido group partially blocked S1 pocket. Senkyunolide A, *Z*-ligustilide, ferulic acid and senkyunolide I were mainly located at S1 pocket by interacting with ASP189, ALA190, CYS191, CYS220, GLY216, GLY219, GLY226, PHE227, TRP215 and so on. Binding energy of them were − 6.48, − 7.13, − 5.3 and − 6.99 kcal/mol, respectively.

**Table 2:** Docking results and residues interactions of four inhibitors screened from Chuanxiong EA fractions and argatroban with THR

Compounds	Docking energy (kcal·mol^−1^)	Hydrogen bond	Van der Waals	Electrostatic interaction and other
Senkyunolide A	− 6.48	–	GLY226, ASP189, TYR225, GLU217, GLY216, GLY219, CYS220, CYS191, SER195, SER214, TRP215, PHE227	VAL213, ALA190, TYR228
*Z*-ligustilide	− 7.13	GLY226, PHE227	TYR225, GLY216, GLU217, ASP221, GLY219, CYS191, GLU192, ASP194, VAL213, TYR228, SER214, TRP215, CYS220	ASP189, LYS224, ARG221A, ALA190
Ferulic acid	− 5.3	SER195, GLY193, ASP189, PHE227, GLY226	TYR225, GLY216, GLY219, CYS191, ASP194, HIS57, GLU192, VAL213, SER214, TRP215	TYR228, ALA190, CYS220
Senkyunolide I	− 6.99	SER195, GLY226	PHE227, TYR225, GLY216, ASP189, GLU217, ASP221, GLY219, ARG221A, CYS191, GLU192, GLY193, ASP194, HIS57, VAL213, TRP215	ALA190, CYS220, SER214, LYS224
Argatroban	− 8.90	GLY219, GLY216, HIS57, SER195, GLY193, ALA190	ASP221, CYS220, CYS191, ASP194, GLU217, TRP215, GLY226, SER214, GLU192, LEU99, CYS42, LEU41, TYR225	TRP60D, ASP189, TYR60A, LYS60F

**Fig. 8:**
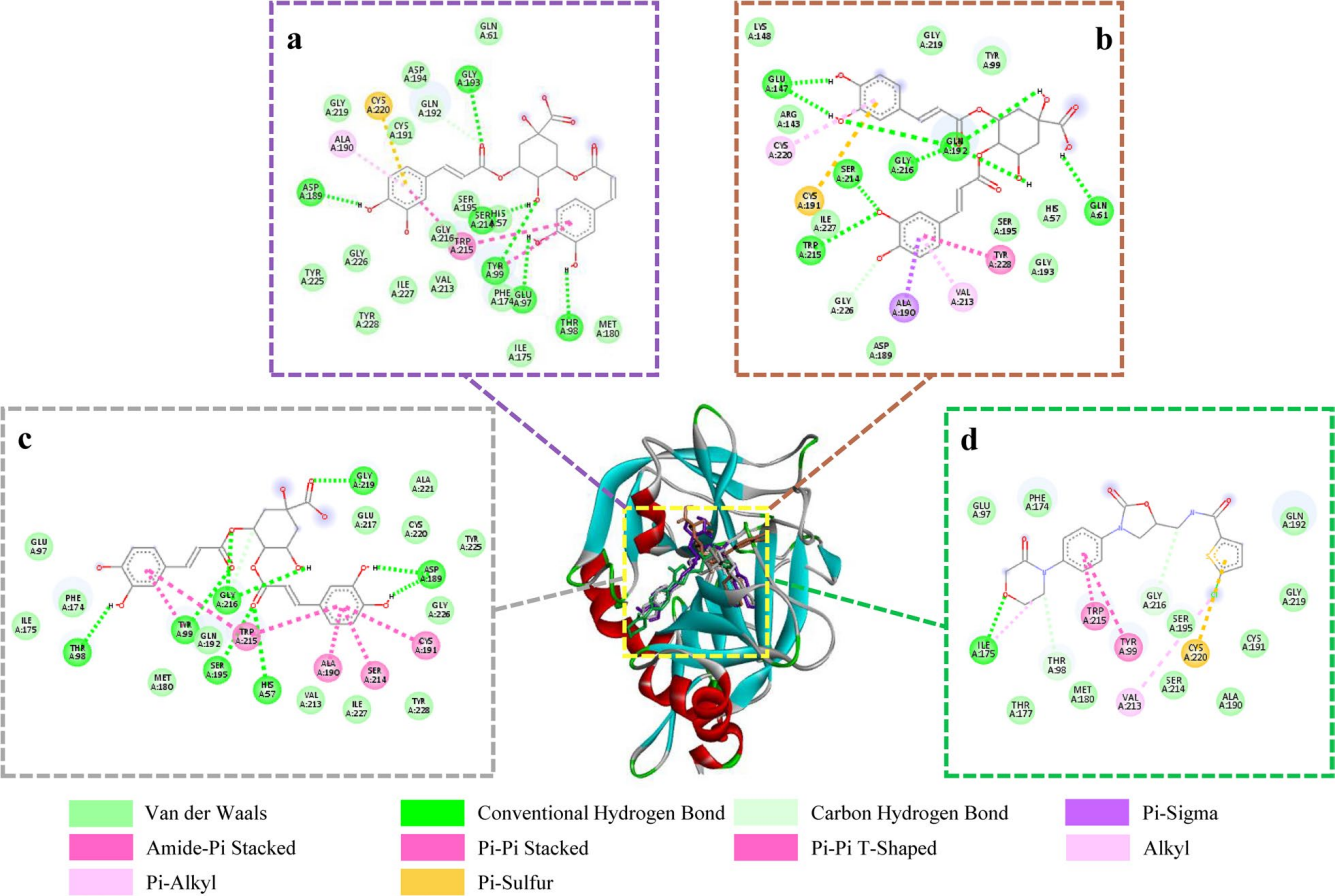
Binding mode and interaction between Isochlorogenic acid A (**a**), Isochlorogenic acid B (**b**), Isochlorogenic acid C (**c**), Rivaroxaban (**d**) and the FXa catalytic site

Molecular docking with FXa was also carried out. The docking energy and binding residues of isochlorogenic acid A (from Chuanxiong BA fractions) and its isomers with FXa active site were shown in Table 3. FXa also has four binding pockets, S1 pocket to S4 pocket [47]. Isochlorogenic acid A, B and C had similar binding portion with rivaroxaban bound to FXa (Fig. 8). The interactions were diverse including hydrogen bond and van der Waals, etc. When docked with FXa, rivaroxaban is mainly located in S4 pocket, and its chlorothiophene amide part interacted with amino acids between S1 and S2 pockets. The caffeinyl group in 5 position of quinine ring of isochlorogenic acid A and C occupied S4 pocket. Another caffeinyl group combined with S1 pocket and penetrated into its bottom through hydrogen bonding with ASP189. The quinine ring of isochlorogenic acid B mainly inserted into S2 pocket, and the caffeinyl groups interacted with the amino acids around S1 pocket. The binding energy of isochlorogenic acid A, B and C were − 9.39, − 8.67 and − 9.71 kcal/mol, respectively.

**Table 3:** Docking results and residues interactions of three isochlorogenic acids and rivaroxaban with FXa

Compounds	Docking energy (kcal·mol^−1^)	Hydrogen bond	Van der Waals	Electrostatic interaction and other
Isochlorogenic acid A	− 9.39	GLN192, GLY193, THR98, GLU97, TYR99, SER214, ASP189	GLN61, MET180, ILE175, PHE174, HIS57, SER195, GLY216, VAL213, ILE227, TYR228, GLY226, TYR225, GLY219, CYS191, ASP194	TRP215, ALA190, CYS220
Isochlorogenic acid B	− 8.67	GLN192, GLN61, TRP215, SER214, GLY216, GLU147, GLY226	HIS57, SER195, GLY193, ASP189, ILE227, ARG143, LYS148, GLY219, TYR99	TYR228, VAL213, ALA190, CYS191, CYS220
Isochlorogenic acid C	− 9.71	GLY216, SER195, HIS57, ASP189, GLY219, THR98, TYR99	GLN192, VAL213, ILE227, TYR228, GLY226, TYR225, CYS220, GLU217, ALA221, GLU97, PHE174, ILE175, MET180	TRP215, ALA190, SER214, CYS191
Rivaroxaban	− 10.19	GLY216, THR98, ILE175	GLU97, PHE174, GLN192, GLY219, CYS191, ALA190, SER195, SER214, MET180, THR177	CYS220, TRP215, TYR99, VAL213

### In vitro activity assessment of the predicted compounds

**Fig. 9:**
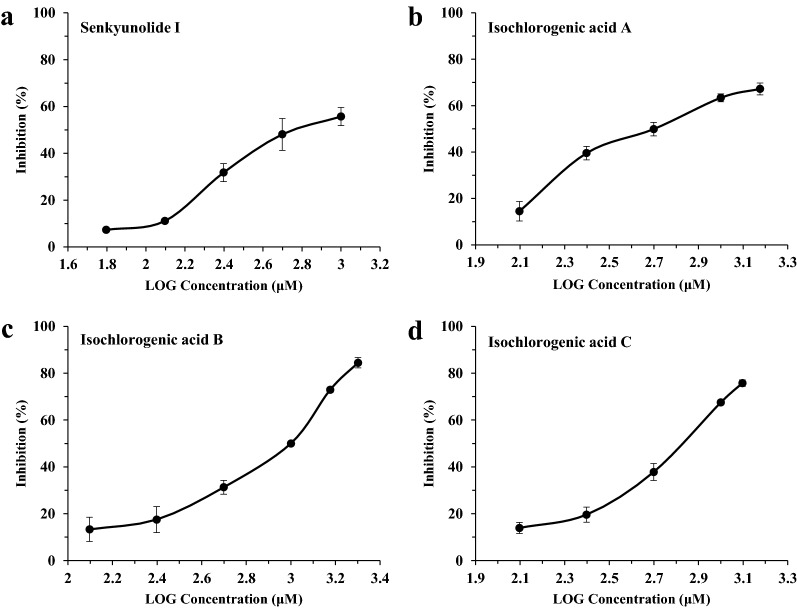
Inhibition effect of Senkyunolide I (**a**), Isochlorogenic acid A (**b**), Isochlorogenic acid B (**c**) and Isochlorogenic acid C (**d**) on enzyme. **a** THR, **b**–**d** FXa. Data are shown as mean ± SD from three independent experiments (n = 3)

The reference compounds, which were based on the four identified markers (senkyunolide A, *Z*-ligustilide, ferulic acid and senkyunolide I) screened from Chuanxiong EA fractions, were further examined for their inhibitory effects on the THR activity. Among them, senkyunolide I strongly inhibited THR activity with an IC_50_ value of 0.77 mM (Fig. 9). Senkyunolide A and *Z*-ligustilide on the inhibiting of THR activity were weaker than that of senkyunolide I with  % inhibition around 40% at a relatively high concentration (0.5 mM), while ferulic acid did not show inhibitory effect on the THR activity under such concentration. The inhibition results were summarized in Table 4. The marker compound screened from Chuanxiong BA fractions, isochlorogenic acid A, and its isomers (isochlorogenic acid B and C) were further examined for their potential inhibitory effects on the FXa activity. From the results shown in Fig. 9, these compounds possessed FXa inhibitory effects in a dose-dependent behavior, and IC_50_ value were 0.56, 0.77 and 0.61 mM, respectively.

**Table 4:** Inhibition effect of Senkyunolide A, *Z*-ligustilide and Ferulic acid on THR

Compounds	Concentration (mM)	Inhibition percentage (%)
Senkyunolide A	0.5	41.90 ± 1.84
	0.25	31.99 ± 2.58
	0.125	28.51 ± 2.42
*Z*-ligustilide	0.5	36.92 ± 2.37
	0.25	26.04 ± 1.84
	0.125	25.52 ± 1.17
Ferulic acid	0.5	− 7.72 ± 1.91
	0.25	1.01 ± 3.59
	0.125	1.01 ± 4.17

## Discussion

Chuanxiong EA extract has the strongest inhibitory activity against THR among four different polar extracts, and its fractions (EA-SC1 to EA-SC5) exhibited activity difference. In order to compare the chemical profiles of five fractions, multivariate statistical analysis with two fraction classes were used. Based on OPLS-DA model, markers **a**–**g** were the main components that contribute to the difference of composition and enzyme inhibitory activity among fractions. Four of them were identified as senkyunolide A (**a**), *Z*-ligustilide (**d**), ferulic acid (**f**) and senkyunolide I (**g**). FXa inhibitory activity assessment results demonstrated a different tendency, in which Chuanxiong BA extracts showed the highest enzyme inhibitory activity. An FXa inhibitor isochlorogenic acid A (**h**) was screened using the same way. Chuanxiong extracts has the different effective position toward THR and FXa, which give a hint that THR inhibitor might mainly exist in low polar phthalides and FXa inhibitors could be found in high polar phenolic acids.

Molecular docking can be used to study the binding mechanism of compounds interact with protein such as characterizing the binding site and evaluating the strength of interaction [48]. It was demonstrated that the four screened marker compounds from Chuanxiong EA extracts were bound to the THR active sites and the binding energies were below − 5 kcal/mol. Generally, the region with binding energy lower than − 5.0 kcal/mol could be considered the “Potential Targets” [49]. Therefore, senkyunolide A, *Z*-ligustilide, ferulic acid and senkyunolide I were potential THR inhibitors. Their inhibitory effects on THR activity were examined. Among them, senkyunolide I had the strongest activity, and senkyunolide A and *Z*-ligustilide showed moderate activity, while ferulic acid exhibited no effect which could explain the low binding energy of ferulic acid that is close to − 5 kcal/mol. In addition, the IC_50_ of senkyunolide I differed from the report [12], which may be due to the different experimental methods and conditions (such as concentrations of substrate and enzyme activity). Likewise, the interaction between isochlorogenic acid A, B and C and FXa were investigated. The result of binding energy of isochlorogenic acid A and C (< − 9 kcal/mol) is better than isochlorogenic acid B (− 8.67 kcal/mol). Isochlorogenic acid A and C interacted with the S1 and S4 pocket of FXa, and isochlorogenic acid B could interact with S1 and S2 pocket. It has been reported that S1 and S4 pockets are commonly used to predict high-affinity FXa inhibitors [50]. Therefore, these three molecules are potential inhibitors of FXa. The results of inhibitory effects on the FXa activity indicated that isochlorogenic acid A and C had the lower IC_50_ value. A possible explanation is that they have similar binding site and energy.

Senkyunolide I, which has been screened out as a THR inhibitor from Chuanxiong ethanol extract by ultrafiltration in our previous study [12], was also screened out in this study along with senkyunolide A and Z-ligustilide. Among the three compounds, senkyunolide I had the strongest inhibitory activity, probably because of its two phenolic hydroxyl groups have hydrogen bonding with SER195 that belongs to the THR catalytic triad. In addition, one of the FXa inhibitors found in this study is isochlorogenic acid C, which was also discovered as a THR inhibitor in our previous study [12]. The explanation may be that its larger molecular structure and more hydroxyl groups make it able to bind to the residues in the active pocket of the center of THR/FXa pocket. Therefore, it could be a dual-enzyme inhibitor.

## Conclusion

Rhizoma Chuanxiong has been used for thousands of years in TCM, and is well-known for its properties of “HuoXueHuaYu” (activating blood circulation to remove blood stasis). It has various kinds of biological activities such as vasodilation, antiinflammatory, antioxidation, antiproliferation, etc. Through the combination method of LC–MS analysis, THR/FXa activity assessment and multivariate statistical analysis, this study predicted and identified four marker compounds (senkyunolide A, *Z*-ligustilide, ferulic acid and senkyunolide I) with potential THR inhibitory activity from Chuanxiong EA fractions, and one marker compound isochlorogenic acid A, with potential FXa inhibitory activity from Chuanxiong BA fractions. Docking results showed that five screened compounds could insert into the catalytic active site of enzyme, and the binding energy was lower than − 5 kcal/mol. The IC_50_ of senkyunolide I and isochlorogenic acid A was 0.77 and 0.56 mM, respectively. In addition, two other FXa inhibitors, isochlorogenic acid B and isochlorogenic acid C, with similar structure to isochlorogenic acid A, were also found, with IC_50_ value of 0.77 and 0.61 mM, respectively. These results proved that the proposed method could effectively characterize the THR/FXa inhibitors in complex mixtures, which not only complemented the anticoagulant mechanism of Rhizoma Chuanxiong, but also provided a clue for the discovery of new active THR and/or FXa inhibitors.

## Data Availability

The research data generated from this study is included within the article.

## Abbreviations

BA: Butanol
CE: Capillary electrophoresis
DAD: Diode array detector
DMSO: Dimethyl sulphoxide
EA: Ethyl acetate
EI: Electrostatic interaction
ESI-MS: Electrospray ionization-mass spectrometer
FXa: Factor Xa
IMER: Immobilized enzyme microreactor
LC–MS: Liquid chromatography paired with mass spectrometry
LGA: Lamarckian genetic algorithm
OPLS-DA: Orthogonal partial least squares discriminant analysis
PCA: Principal component analysis
PE: Petroleum ether
UPLC-QTOF-MS: Ultra performance liquid chromatography-quadrupole-time of flight-mass spectrometer
RE: Remained extract
SC: Silica gel column chromatography
TCM: Traditional Chinese medicine
THR: Thrombin
Tris: Tris (hydroxymethyl) aminomethane
VIP: Variable importance in the projection
WE: Water extract
